# Evaluating the role and integration of general practice pharmacists in England: a cross-sectional study

**DOI:** 10.1007/s11096-021-01291-6

**Published:** 2021-06-02

**Authors:** Abdullah A. Alshehri, Ejaz Cheema, Asma Yahyouche, M. Sayeed Haque, Zahraa Jalal

**Affiliations:** 1 School of Pharmacy, College of Medical and Dental Sciences, University of Birmingham, Edgbaston, Birmingham, B15 2TT UK; 2 Clinical Pharmacy Department, College of Pharmacy, Taif University, Al Huwaya, Taif, 26571 Saudi Arabia; 3 Institute of Applied Health Research, College of Medical and Dental Sciences, University of Birmingham, Edgbaston, Birmingham, B15 2TT UK

**Keywords:** England, General practice, Health services, Integration, Intervention, Pharmacist

## Abstract

*Background* Since 2015, NHS England has facilitated the recruitment of pharmacists in general practice (GP) to reduce workload of general practitioners. The role of pharmacists is therefore expected to become more clinical and patient oriented. However, little is known about the current roles performed and the integration of GP pharmacists. *Objective* To assess the role performed by GP pharmacists and their integration into practice exploring facilitators and barriers to integration. *Setting* A cross-sectional survey of GP pharmacists in England. *Method* This study used both online and paper-based questionnaires for a period of six months. Survey items included demographics, roles performed, integration including available support and practice environment. Quantitative data were analysed using descriptive statistics and 95% confidence intervals. Open comments were analysed thematically to identify pharmacists’ perceptions of barriers and facilitators to their integration into practice. *Main outcome measure* Current role and integration of pharmacists into GP. *Results* 195 participants completed the questionnaire. Three quarters of pharmacists (76%) had only been in GP since 2015. Most pharmacists (81%) were independent prescribers (PIPs). The most reported pharmacists’ roles were medicine reconciliation (95%), telephone support for patients (95%) and face-to-face medication review (91%). 82% (95% CI: 76% to 86.8%) were satisfied with their overall integration into practice. Half of pharmacists (45%) were working in a shared office or at a hot desk and 9% had no designated workspace. PIPs had more access to a convenient workplace (*p* = 0.016) compared to non-IPs. *Conclusion* Practice pharmacists are fulfilling a wide range of clinical and non-clinical roles in England. Findings highlight relatively a satisfactory level of pharmacists’ integration into practice and shed the light on their integration issues. These findings could be significant for the development of future roles of pharmacists in GP.

## Impacts on practice

- The findings of this study suggest that pharmacists’ led clinical roles including face-to-face medication reviews, medicine reconciliation and telephone support for patients have clearly evolved across the general practice in England
- Pharmacists identified supportive practice and the presence of an experienced pharmacist in the workplace as facilitators to integration into the general practice
- Lack of understanding of the pharmacists’ role by the practice team, contrasting cross-sector professional experience, lack of structured training and supervision were identified as barriers to pharmacists’ integration into practice

## Introduction

The increasing number of patients with multimorbidity and the associated increase in medication use has led to a significant increase in the workload of general practice (GP) worldwide [1–3]. Furthermore, shortfalls in the recruitment and retention of general practitioners coupled with their early retirement have exacerbated the workload issues [4]. Pharmacists, considered as qualified experts in medicines with range of knowledge and clinical skills, are expected to undertake a range of activities in GP such as medication reviews and management of both minor ailments and long-term medical conditions [5, 6]. Evidence suggest that pharmacists can provide valuable services to ease the burden on GP and reduce patient waiting times [6–8]. Moreover, integrating pharmacists into GP could reduce emergency department attendance and improve patient safety and health outcomes [9, 10]. Evidence from published international systematic reviews and meta-analysis of randomised control trials suggests that pharmacists who are working in GP can significantly improve clinical outcomes of patients with chronic diseases [11, 12].

Several studies conducted in the United Kingdom, Australia and Canada suggest that pharmacists’ role within GP have developed and evolved recently towards a more clinically oriented role [13–15]. In 2015, the National Health Service England (NHSE) launched the ‘Clinical pharmacists in General Practice’ pilot scheme which started with funding 490 clinical pharmacists (CPs) for supporting the management of long-terms conditions and improving patients experience in GP settings [16, 17]. The pilot scheme (phase 1) has been associated with a significant reduction in the workload and improvement in the capacity and quality of patient care in GP [17]. These findings have encouraged the government to invest over £112 million of funding to general practices to recruit and train an additional 1500 CPs by 2020–2021 [17, 18]. The scheme has supported pharmacists by providing funding for independent prescriber (IP) courses and national training and education pathways to help them deliver patient-facing clinical roles in GP including a range of enhanced services and prescribing [18–20]. Further investment is planned for Primary Care Networks (PCNs) to substantially expand the number of pharmacists to as high as 7500 by 2024 [21–23]. Pharmacists will support PCNs with directed enhanced service delivery, the quality and outcomes framework (QOF), and other national and local incentive schemes [22]. Consequently GPs and PCNs would be expected to devote more time and resources in developing their pharmacists to get the best return on investment [24].

To date, two studies suggest that the GP pharmacists recruited through the NHS pilot scheme were reportedly satisfied with their integration into practice and were involved in the delivery of a wide range of clinical and non-clinical activities [13, 17]. However, little is known about the activities and integration of pharmacists who have been recruited into the GP outside the pilot scheme [25]. A recent Scottish survey reported the perspective of practice-based pharmacists about their role and integration [26]. However, the findings of this study may not be generalisable to England where established services and funding bodies are different [16]. There is a need therefore, to explore the role undertaken by GP pharmacists and their integration into practice across England regardless of the scheme, including activities undertaken, scope of practice, working status, perception of integration and available support as well as practice environment.

### Aim of the study

This study aimed to assess the role performed by GP pharmacists and their integration into practice exploring facilitators and barriers to integration across England.

### Ethics approval

Ethical approval was obtained from the ethics committee at University of Birmingham (Reference Number: ERN_18-0859).

## Method

### Design and setting

A cross-sectional survey using a self-administered questionnaire was conducted for a period over six months between May and November 2019.

### Questionnaire development

The questionnaire was developed using a previously validated survey tool to assess the role and integration of pharmacists into GP practice [13]. It consisted of 35 questions that included both open-ended and closed questions and was formatted in a multiple-choice and 5-point Likert scale format (where 1 = not at all satisfied to 5 = very satisfied). The questionnaire included four major domains: (1) demographics such as age, gender and previous experience (duration and sector), (2) current GP pharmacists’ role, (3) satisfaction with integration into practice and available support from practice team, (4) practice environment. Clinical and organisational integration was assessed via pharmacists’ perceptions about their integration into practice and support received from general practitioners and other practice team members. Practice environment was evaluated by considering: number of practice meetings attended, formal appraisal conducted and type of physical workspace. The questionnaire was expected to take around 10–15 min to complete.

### Piloting the questionnaire

The paper version of the questionnaire was piloted by seven practice pharmacists to check the readability, understanding and feasibility of the questionnaire. Based on the pharmacists’ feedback, one question that was not related to the study aims was removed from the questionnaire. Also, some minor changes were made to the wording of items. The questionnaire was then set up as an online survey and was further piloted by 10 practice pharmacists to pilot the online version for administration purposes.

### Data collection

A convenience sample of GP pharmacists was constructed for the study. Both paper and online versions of the questionnaire were used to maximise the number of the participants. Paper version was distributed to participants during pharmacy training and professional events. An online version was distributed through a link to social media platforms (Twitter, LinkedIn and Telegram) and direct emails to gatekeepers at Midlands Practice Pharmacy Network.

### Data analysis

The data from both paper and online questionnaires were analysed using statistical package SPSS (version 26). Descriptive statistics were used to summarise the quantitative data. The difference in proportions (diff) between Pharmacist Independent Prescriber (PIPs) and non-Independent Prescribers (non-IPs); with their 95% confidence intervals (CI) as recommended by the Newcomb 1998 [27] were calculated. Fisher’s exact test was also performed to identify associations of performed roles and integration into practice including pharmacists’ scope of practice, integration into practice, practice environment and demographic factors such as level of experience between PIPs and non-IPs. Statistical significance was assessed at *p* < 0.05. Thematic analysis [28] was used to analyse open-ended question responses. Coding was carried out using an inductive approach which involved grouping similar comments of text and allocating labels or “codes”. These were then grouped into two higher level themes defined as “pharmacists’ barriers to integration”, and “pharmacists’ facilitators to integration”. The analysis involved an iterative process of allocating text to codes or sub-themes, and then reviewing these, reallocating text between them, and re-labelling the codes until the final distribution of data by themes was deemed to most accurately reflect and convey the overall findings. This process was performed manually by two researchers (AA and AY) independently and the results were discussed with the research team to reach a consensus on the themes and codes.

## Results

### Characteristics of respondents (n = 195)

Table 1 shows the characteristics of the participants. Survey responses were received from 195 practice pharmacists in England. Of these, 89 were completed on paper and 106 were online. Most participants (n = 176, 90%, 95% CI: 85–93.6%) had been registered as pharmacists in England for 6 years or more; the majority (n = 148, 76%, 95% CI: 69–81%) had been in GP for 4 years or less. Regarding cross-sector professional experience, almost two-thirds (n = 117, 60%, 95% CI: 53–66.6%) had worked previously in community pharmacies, 10% (n = 20, 95% CI: 6.7–15%) had worked in hospitals and 9% (n = 18, 95% CI: 5.9–14%) had worked in both sectors. Moreover, 81% (n = 158, 95% CI: 75–86%) were PIPs, most of them (n = 98, 62%, 95% CI: 54–69%) received their independent prescriber (IP) qualification in the last 3 years. Most PIPs (n = 139, 88%, 95% CI: 82–92%) scope of practice was in long-term medical conditions mainly hypertension, diabetes or respiratory diseases. Most PIPs were currently prescribing (n = 151, 95%, 95% CI: 91–97.8%), with variation in the number of items prescribed weekly.

**Table 1:** Characteristics of participants

Characteristic	N (N = 195)	%
Gender		
Female	122	62.6
Male	72	36.9
Other	1	0.5
Age		
Range = 36, Median = 39 (IQR = 13)		
Years since posted in general practice		
≤ 1–4 years	148	75.9
5–10 years	21	10.7
11–15 years	11	5.6
≥ 16 years	15	7.7
Cross-sector professional experience		
Community pharmacy	117	60.0
Hospital pharmacy	20	10.2
Mixed of community and hospital	18	9.2
Primary care organisation (e.g. CCG, PCT)	13	6.6
Mixed of other sectors with General practice	6	3.0
Other or mixed sector	21	10.7
Level of experience		
Senior pharmacists	60	31.7
Pharmacists posted through the NHSE Scheme	96	49.2
Pharmacist Independent Prescriber (PIPs) (N = 158)		
Conditions qualified in as PIPs		
Hypertension	67	42.4
Diabetes	22	13.9
Respiratory (Asthma or COPD)	21	13.2
Generalist	19	12.0
Other	29	18.3
Level of experience as PIPs		
≤ 1–3 years	98	62.0
> 4 years	60	37.9

### Pharmacists’ roles (scope of practice and working status)

**Fig. 1:**
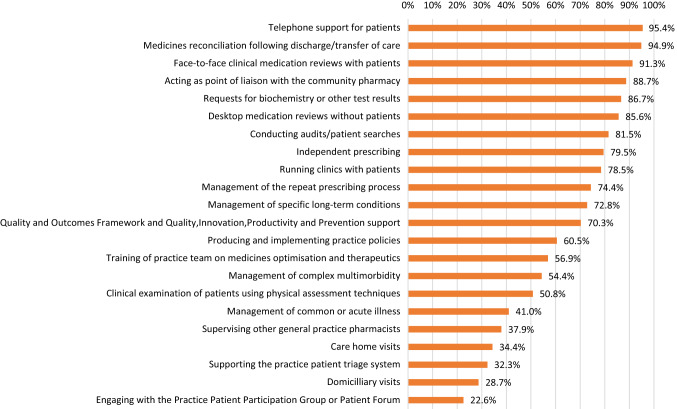
Activities performed by general practice pharmacists in England. QIPP = Quality, Innovation, Productivity and Prevention. QOF = Quality and Outcomes Framework. Note; descriptive statistics were employed to derive percentages. Results were retrieved regular weekly basis activities

Figure 1 presents the activities performed by GP pharmacists in England. The most commonly reported roles included telephone support for patients (n = 186, 95%, 95% CI: 91–97.5%), medicine reconciliation following discharge/transfer care (n = 185, 95%, 95% CI: 90.8–97%) and medication reviews with patients (n = 178, 91%, 95% CI: 86.5–94.5%). 52% (n = 101, 95% CI: 45–59%) of pharmacists worked part-time and 48% (n = 94, 95% CI: 41–55%) worked full-time in GP. With regards to the range of number of practices, 54% (n = 105, 95% CI: 47–61%) of pharmacists were based in one practice, while 46% (n = 90, 95% CI: 39–53%) were based in two or more practices. Pharmacists indicated that they were working 6–50 h per week in GP, with a median of 32 h (Interquartile Range = 15). Patient-facing roles were either face-to-face (median = 8 h, Interquartile Range = 13) or by telephone (median = 4, Interquartile Range = 8). A quarter (n = 47, 24%, 95% CI: 19–31%) of GP pharmacists were exclusively treating patients with specific conditions mainly hypertension and diabetes but the remaining (n = 148, 76%, 95% CI: 69–81%) dealt with any clinical condition that came to their practice.

### Integration into practice and support

**Fig. 2:**
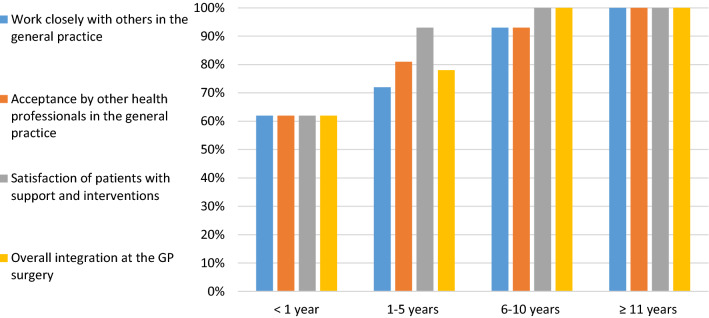
Pharmacists’ satisfaction about their integration over time working in general practice

A total of 82% (n = 160, 95% CI: 76–86.8%) of pharmacists reported they were satisfied (either very satisfied or satisfied) with their overall integration into practice (Table 2). Pharmacists reported limited satisfaction in relation to their integration when working with the other members of the multidisciplinary team (n = 149, 76.5%, 95% CI: 67–81.8%). In particular they were less satisfied with the support received from general practitioners in specific (n = 151, 77.5%, 95% CI: 71–82.7%) and from other practice staff (n = 155, 79.5%, 95% CI: 73–84.5). However, the satisfaction levels on the assessed items of integration into practice were found to be the lowest (60%) in pharmacists with less than one year’s experience in GP, and this gradually increased with an increased number of working years (Fig. 2).

**Table 2:** Pharmacists' satisfaction about their integration and available support

Statements	Number of VS or S (%)	95% CI
Work closely with others in GP	149 (76.4)	69.98–81.82
Acceptance by other health professionals in GP	163 (83.6)	77.75–88.13
Patients’ satisfaction with pharmacist support and interventions	183 (93.8)	89.55–96.45
Overall integration at the GP surgery	160 (82.0)	76.06–86.80
Support received from general practitioners colleagues	151 (77.5)	71.07–82.74
Support received from other practice staff e.g. nurses and clerks etc	155 (79.5)	73.28–84.56

*VS* very satisfied, *S* satisfied, *CI* confidence interval, *GP* general practice

### Practice environment

Fifty-four percent of participants (n = 105, 95% CI: 46.8–60.7%) had attended half or more of the practice's meetings, 33% (n = 65, 95% CI: 27–40%) had attended less than 50% and the remainder 13% (n = 25, 95% CI: 8.8–18%) had not attended any practice's meeting. Half of respondents (n = 110, 56%, 95% CI: 49–63%) had received a performance appraisal; 11% (n = 22, 95% CI: 7.5–16%) had one arranged but not yet conducted; 7% (n = 13, 95% CI: 4–11%) had been offered an appraisal but it had not been arranged; and the remaining 26% (n = 50, 95% CI: 20–32%) had not yet been offered any appraisal. Appraisals were conducted mostly by the GP clinical supervisor (n = 71, 57.7%, 95% CI: 49–66%) followed by the senior pharmacist (n = 26, 21%, 95% CI: 14.8–29%), practice manager (n = 17, 14%, 95% CI: 8.8–21%) and then other general practitioners in the practice (n = 9, 7.3%, 95% CI: 4–13.3%). Regarding GP pharmacists’ workspace, 46% (n = 89, 95% CI: 38.8–52.6%) were working in a private room, 32% (n = 63, 95% CI: 26–39%) were working in a shared office with practice colleagues, 13% (n = 26, 95% CI: 9–18.8%) were working at a hot desk, and the remaining respondents (n = 17, 9%, 95% CI: 5.5–13.5%) had not been provided with a designated workspace. Of the pharmacists sharing an office with colleagues, 35% (n = 22, 95% CI: 25.7–45%) shared it with practice administration, 26% (n = 16, 95% CI: 17.8–35.8%) with other pharmacists, 22% (n = 14, 95% CI: 15–32%) with general practitioners, 11% (n = 7, 95% CI: 6–19%) with nurses, and 6% (n = 4, 95% CI: 2–12.5%) with pharmacy technicians.

### Comparison between PIPs and non-IPs

There was a significant association in over half of the evaluated roles between PIPs and non-IPs (Table 3) such as running clinics (*p* < 0.001, diff = 36.8%, 95% CI: 20–52.7%), clinical examination of patients using physical assessment techniques (*p* = 0.002, diff = 29%, 95% CI: 11.5–43%) and medication reviews with patients (*p* = 0.005, diff = 16%, 95% CI: 4.6–31.7%). There was no difference between the two groups in roles such as liaison with the community pharmacy (*p* = 1.000, diff = −0.59%, 95% CI: −9.4–13.9%), conducting audits/patient searches (*p* = 1.000, diff = 0.5%, 95% CI: −11.05–16.7%) and care home visits (*p* = 1.000, diff = −1%, 95% CI: −18.5–14.4%).

**Table 3:** The significant difference between the roles performed by PIP and non-IP pharmacist in general practice

Pharmacists’ roles at GP surgery	PIPs (%)	Non_ IPs (%)	*P* value
Running clinics with patients	135 (85.4)	18 (48.6)	< 0.001
Supervising other general practice pharmacists	71 (44.9)	3 (8.1)	< 0.001
Clinical examination of patients using physical assessment techniques	89 (56.3)	10 (27.0)	0.002
Management of complex multimorbidity	94 (59.5)	12 (32.4)	0.003
Face-to-face clinical medication reviews with patients	149 (94.3)	29 (78.3)	0.005
Producing and implementing practice policies	103 (65.1)	15 (40.5)	0.008
Training of practice team on medicines optimisation and therapeutics	97 (61.3)	14 (37.8)	0.010
Management of the repeat prescribing process	124 (78.4)	21 (56.7)	0.011
Management of specific long-term conditions	121 (76.5)	21 (56.7)	0.023
Management of common or acute illness	71 (44.9)	9 (24.3)	0.026

*P* value calculated by using Fisher’s Exact Test (FET) where *P* value < 0.05 is statistically significant

The analysis also reported that PIPs had more access to work in a private room (*p* = 0.016, diff = 23%, 95% CI: 5–37%) and to conduct a formal appraisal or review of their progress (*p* < 0.001, diff = 33%, 95% CI: 15–47%) which was statistically significantly different compared to non-IPs. However, there was no significant difference between the groups in attending of the practice's meetings (*p* = 0.36, diff = 3.87%, 95% CI: −12.5–15.7%). Moreover, there was a significant difference in the satisfaction of PIPs, who perceived that patients were satisfied with their support and interventions (*p* = 0.012, diff = 12%, 95% CI: 2.8–27%) compared to non-IPs.

### Open comments

A total of 124 open comments related to the barriers and facilitators to the integration of pharmacists into practice were submitted. Table 4 shows the generated themes of barriers and facilitators to the integration of GP pharmacists with supporting quotations.

**Table 4:** Identified barriers and facilitators to the pharmacists’ integration into general practice with supporting quotations

	Themes	Supporting quotations
Barriers of integration into practice	Practice teams’ lack of understanding of the pharmacist role	*“GPs still struggling to grasp what exactly pharmacist do”* (Pharmacist No. 7) *“staff don’t fully understand the role of clinical pharmacist”* (Pharmacist No. 50)
	Lack of clinical support from practitioners	*“Role can still be quite isolating as you can be the only one in a practice.”* (Pharmacist No. 51) *“…GPs and a nurse practitioner have been less supportive and criticised the role”* (Pharmacist No. 91)
	Policy/funding-related	*“Difficult with NHSE funding that was spread so thinly. The PCN's are also at risk of doing the same, replacing pharmacists across several sites to do projects- likely to have an impact on the 'practice pharmacist' elements if we are not there as much”* (Pharmacist No. 3) *“There is a pressure to work as quick as the GPs, especially when dealing with repeat prescribing issues and medication queries. This is only possible when there are clear policies and procedures outlined in the surgery “* (Pharmacist No. 58)
	Unreasonable expectations	*“I am doubting my own self confidence … struggle as first role as IP”* (Pharmacist No. 74) *“My original area was diabetes. I have now extended my competencies beyond this …”* (Pharmacist No. 49) *“… staff assume can sign all scripts but I don't”* (Pharmacist No. 32)
	Workloads and time constraints	*“… barriers to integration is 3 practices for half day a week”* (Pharmacist No. 42) *“Not properly integrated Massive workload”* (Pharmacist No.44)
	Lack of adequate training and professional experience	*“No structured training…"* (Pharmacist No. 36) *“It is a steep learning curve when switching from community to GP practice. So much more clinical need to learn”* (Pharmacist No. 32)
Facilitators of integration into practice	Supportive practice	*“…felt welcomed and part of the team …”* (Pharmacist No. 13) *“My practice manager was key to my integration and advocate for the role.”* (Pharmacist No. 40)
	Adequate training/supervision	*“I believe that clinical supervision and experiential learning across the MDT is a must.”* (Pharmacist No. 19)
	Pharmacist presence in GP	*“There was already a practice pharmacist when I joined so this made my intervention somewhat easier"* (Pharmacist No. 17)
	Stability or continuity	*“It has evolved vastly in the two years I have been in post as my, and the team's, confidence has grown in me.”* (Pharmacist No. 31) *“Working in one practice is much better than trying to spread my skills over a number of practices.”* (Pharmacist No. 14)
	Pharmacists own attitude/approach	*“Practice pharmacists have to integrate themselves into an MDT and quickly assess where the need is for them to make the biggest impacts. They don't necessarily know the wide and significant impacts pharmacists can make, we have to prove it to them.”* (Pharmacist No. 96)
	Patient awareness and understanding	*“I've been in GP surgeries since 2012, things have changed so much in this short time, in terms of attitude towards pharmacists both by patients and staff”* (Pharmacist No. 86)

## Discussion

This study investigated the current role of GP pharmacists and their integration including the types of undertaken activities, scope of practice and working status, perception of integration and available support in addition to the practice environment. The findings of this study suggest that clinical roles have clearly evolved across the GP in England with face-to-face medication reviews, medicine reconciliation and telephone support for patients as main roles. However, this study highlighted issues about the practice environment, the levels of satisfaction with regards to feeling integrated when working as part of a multidisciplinary team and with the available support from practice team.

Evidence suggests that medication reviews and medicines reconciliation are two important activities undertaken by pharmacists to improve the clinical, economic and humanistic outcomes of patients [11, 29–31]. Within the various clinical activities undertaken by GP pharmacists across England, medication reviews and medicine reconciliation remained as major part of their roles. These findings are consistent with the previous studies that evaluated pharmacists’ role within the pilot scheme [13, 17] or across the UK [25, 26]. A recent review from multiple countries and contexts conducted to inform the development of a comprehensive role description for practice pharmacists has detailed seven role sub-categories (medication management, patient examination and screening, chronic disease management, drug information and education, collaboration and liaison, audit and quality assurance and research) [32]. Over 80% of responding pharmacists were IPs with scope of practice mainly in hypertension, diabetes and respiratory diseases similar to other studies [17, 25, 26]. This may reflects the significant funding provided for pharmacists across the UK to be prescribers and practice in the primary care settings [18, 33]. In Scotland, for example, the future plan of pharmaceutical care expects all pharmacists working in either GP or community pharmacies to become IPs by 2023 [34]. However, PIPs in this study reported issues and concerns about providing roles outside their clinical competence [35]. The findings of this study suggest the need to extend the current scope of IP course curriculum to other long-term medical conditions with complex co-morbidities to develop pharmacists' confidence outside their scope of expertise to better match the expectations of GP.

Bradley et al. investigated the integration of pharmacists recruited through the pilot scheme and discussed some issues with the physical workspace and the level of support from general practitioners [13]. This is consistent with the findings of this study that included all GP pharmacists in the study. These issues have also been identified as major barriers to integration of nurse and pharmacist practitioners in Australia and Canada [36–38]. Open comments analysis in this study also highlighted other barriers to pharmacists’ integration including lack of understanding of the pharmacist role by practice team, contrasting cross-sector professional experience, lack of structured training and supervision and time constraints in GP. On the contrary, supportive practice and the presence of an experienced pharmacist in the workplace were identified as facilitators to integration into GP. Besides England [17], similar findings have also been reported by other studies involving different international healthcare systems including Canada [37, 39], Australia [14] and New Zealand [40].

A number of policies has been introduced recently in the UK and not just in England to expand the current involvement of clinical pharmacists in general practice [18, 34, 41]. The findings of this study provide important insights to policymakers and practice managers into the support required by GP pharmacists to deliver a wide range of patient oriented services. Moreover, the success of the new model, PCNs, shall largely depend on what it could potentially learn from the current practice to ensure the efficient and smooth integration of GP pharmacists. The findings of this study suggested that a successful integration would require both pharmacists and the practice team to understand each other's roles well besides receiving structured training to ensure the provision of effective pharmacists-led patient care. However, integration could be challenging for those pharmacists with limited experience in GP. Furthermore, our findings are similar to another study that reported the need to develop the skills and knowledge of new pharmacists in GP setting to perform their roles [42]. The General Pharmaceutical Council has recently implemented new standards for the initial education and training of pharmacists across the UK to equip those providing further clinical roles including independent prescribing from the point of registration [43].

The study has some limitations. Due to the use of convenience sampling in the study, the findings of the study may not be generalisable to the wider population. Furthermore, the use of self-reported responses to the questionnaire may be associated with validity issues. The use of convenience sampling together with self-reported answers suggest that findings of this study should be interpreted with caution. Another limitation relating to the comparison between PIPs and non-IPs is that the number of participants was not equal in two groups. Although the questionnaire used both closed and open-ended questions, there was limited qualitative data which may not be detailed enough to provide an in-depth analysis of pharmacists’ barriers and facilitators to the integration into practice. Therefore, the qualitative findings of this study would need to be confirmed by conducting a further qualitative interview-based study. Future research should include in-depth qualitative interviews with key stakeholders from GP practice to provide a detailed insight about barriers to integration.

## Conclusion

This study provides insight into the current role and integration of GP pharmacists in England. The study suggests that pharmacists are delivering a wide range of patient-oriented services that are a mix of administrative and clinical face to face patient roles. They are faced with challenges related to working environment as well as available support. These factors are crucially important when developing GP pharmacists’ role and need to be considered to ensure the appropriate use of pharmacists’ knowledge and clinical skills and the useful contribution of pharmacist in tackling the shortfalls in the general practitioners’ recruitment and easing the GP workload.
